# Physiological Signatures of Changes in Honeybee’s Central Complex During Wing Flapping

**DOI:** 10.1093/jisesa/ieac060

**Published:** 2022-10-12

**Authors:** Haojia Ding, Shaoze Yan

**Affiliations:** State Key Laboratory of Tribology in Advanced Equipment (SKLT), Division of Intelligent and Biomechanical Systems, Department of Mechanical Engineering, Tsinghua University, 100084 Beijing, China; State Key Laboratory of Tribology in Advanced Equipment (SKLT), Division of Intelligent and Biomechanical Systems, Department of Mechanical Engineering, Tsinghua University, 100084 Beijing, China

**Keywords:** central complex, local field potential, wing flapping, rhythm, brain activity

## Abstract

Many kinds of locomotion abilities of insects-including flight control, spatial orientation memory, position memory, angle information integration, and polarized light guidance are considered to be related to the central complex. However, evidence was still not sufficient to support those conclusions from the aspect of neural basis. For the locomotion form of wing flapping, little is known about the patterns of changes in brain activity of the central complex during movement. Here, we analyze the changes in honeybees’ neuronal population firing activity of central complex and optic lobes with the perspectives of energy and nonlinear changes. Although the specific function of the central complex remains unknown, evidence suggests that its neural activities change remarkably during wing flapping and its delta rhythm is dominative. Together, our data reveal that the firing activity of some of the neuronal populations of the optic lobe shows reduction in complexity during wing flapping. Elucidating the brain activity changes during a flapping period of insects promotes our understanding of the neuro-mechanisms of insect locomotor control, thus can inspire the fine control of insect cyborgs.

Wing flapping is a general flight mode of insects, and its generating process and control mechanism has received a great deal of interest. From the aspects of aerodynamics and mechanism theories, some novel designs of bionic micro-aerial vehicles (MAVs) and aerospace vehicles were inspired by insects’ flapping behavior (Sane 2003, Pines and Bohorquez 2006). By imitating the flapping-wing manner of neopteran flying insects, the microrobots powered by soft artificial muscles achieve controlled flight (Chen et al. 2019). The design of deployable skeleton structures that can be applied to the morphing nose cone is inspired by variable geometry mechanism of the honeybee abdomen (Zhang et al. 2019, 2022). Also, some novel biological structures such as the folded intersegmental membrane of the honeybee abdomen are discovered to apply to the design of bionic mechanical structures (Zhao et al. 2016, Liang et al. 2019).

Animal movement emerges from the complex interplay among many parts such as the brain, muscle, and environment (Nishikawa et al. 2007). For example, the flight behavior of insects is a complex movement that is jointly regulated by various neural pathways such as visual, tactile, olfactory pathways, etc. ([Bibr CIT0039][Bibr CIT0043], Baird et al. 2006, Budick and Dickinson 2007, Srinivasan 2011). The brain is the central part of motor control, however, the detailed roles of its specific subregions in the emergence of wing flapping are not very clear. Although coordination functions of visual systems etc. during flight have been researched a lot, there is plenty of room to explore the brain regions directly related to flight and real-time brain activity change patterns.

It was found that the central complex (CX) played a significant role in insect locomotion, including providing calculation functions in spatial orientation memory (Neuser et al. 2008), position memory (Ofstad et al. 2011), angle information integration (Green et al. 2017, Turner-Evans et al. 2017), polarized light guidance, etc (Heinze and Homberg 2007, El Jundi et al. 2015, Pegel et al. 2018). CX features prominently in the visual navigations of some insects’ flight, such as visual information processing in the honeybee (Apis mellifera, L) (Homberg 1984, Milde 1987) and flight guided by polarized light in the cockroach (Blaberus discoidalis) and locust (Schistocerca gregaria), etc. (Heinze and Homberg 2009, Heinze et al. 2009, Zeller et al. 2015, Held et al. 2016). Recent works show that CX plays a fine-tuning role in many tasks of motion control, especially the onset-offset control of complex tasks and the regulating functions in time structures and amplitudes of locomotion in the cockroach *Blaberus discoidalis* and *Drosophila* (Ridgel et al. 2007, Kahsai et al. 2010, Pfeiffer and Homberg 2014). In addition, the comprehensive processing of multiple sensory information during locomotion decisions might be related to the activities of CX in the cockroach *Blaberus discoidalis* and *Drosophila*, etc. (Pick and Strauss 2005, Ritzmann et al. 2008, Bender et al. 2010). Behavioral experiments also show that insects make locomotion decisions with the participation of CX (Ritzmann et al. 2012). If the CX is damaged by a specific drug, the cockroach is not able to make locomotion decisions such as obstacle avoidance (Pollack et al. 2011). Expect for CX, the posterior median protocerebrum (PMP) is thought to be involved in flight control. There are neural connections between PMP and the descending interneurons that transmit information to motor neurons that control the muscle groups of the wings and neck in the honeybee *Apis melifera* (Goodman et al. 1987).

To explain a specific behavior of insects, it is an effective way to collect the electrophysiological signals for analysis (Horcholle-Bossavit et al. 2007, Hu et al. 2019, Zhao et al. 2020, Ding et al. 2021). Here, we analyzed the local field potentials (LFPs) in the CX of honeybees during flapping and obtained the rhythmic changes and nonlinear dynamic changes of neural activities. By analyzing the LFP signals during flapping, changes in the neural activity patterns were explored, and part of the neural basis of wing flapping was revealed.

## Material and Methods

### Animals

Foraging workers (*Apis melifera carnica*) were captured from honeybee hives (temperature: 25°C, humidity:55%) in the bionic lab of Tsinghua University (116.33°E, 40.00°N). The captured honeybees were in good health and were raised in bottles with sucrose solution.

### Animal Preparation

**Fig. 1.**
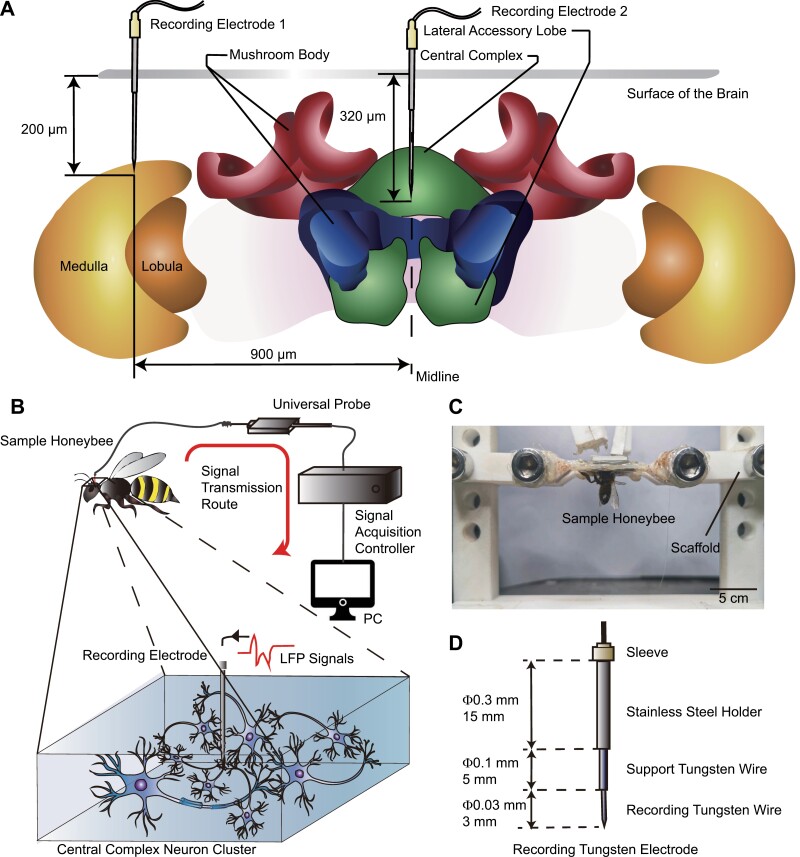
Experimental procedures and the recording electrode. (A) The implantation position and depth of recording electrodes. (B) LFP Signals recording procedures. LFP signals of CX and OL were recorded by the recording electrode, then transmitted via the universal probe and the signal acquisition controller, and finally saved in PC. (C) The sample honeybee was immobilized on the scaffold for anatomy. (D) The dimensions of the recording tungsten electrode that is suitable for the brain of honeybee. The recording tip of the electrode is a tungsten wire with a diameter of 0.03 mm and a length of 3 mm.

The honeybees were frozen at −25°C for 3 min for anesthesia. The sample honeybee after anesthesia was restrained to the scaffold using melted beeswax. There was a gap at the central axis of the scaffold for restraints that was equivalent to the width of the honeybee’s neck, which could fix the honeybee’s head firmly. On this occasion, as shown in Fig. 1B, only the head of the honeybee was exposed above the scaffold, and the thorax and the abdomen were both under the scaffold. The exoskeleton of the honeybee’s head along the edges of two compound eyes, the ocellus, and antennal fossa was removed, thus the brain was exposed completely. Then, to expose the brain surface, the glands and trachea were carefully removed.

### Signal Acquisition

The honeybee that was immobilized on the scaffold was placed on the platform of the brain stereo-positioner (ITEM51900, Yuyan, Shanghai, China). The tungsten wire electrode (Kedou, Suzhou, China) was fixed on the arm of the brain stereo-positioner, then implanted into the central body of CX of the honeybee. The structure and dimensions of the recording electrode are shown in Fig. 1C. The positional data refer to Zhao et al. (2014). In addition, in order to find the position in the dorsoventral direction, the landmark of the α-lobe was used to assist in localization. The transparent mango-shaped regions in the middle of the honeybee brain indicate the position of the α-lobes (Zhao et al. 2014). In the process of electrode implantation, saline solution (0.130 mmol/L NaCl, 0.006 mmol/L KCl, 0.004 mmol/L MgCl_2_, 0.005 mmol/LCaCl_2_, 0.010 mmol/L HEPES, 0.025 mmol/L Glucose, 0.160 mmol/L Sucrose) was added to the surface of the brain to keep it active. In the experiment, the signal transmission route is shown in Fig. 1A. The collecting electrode was connected to the universal probe (Syntech, Germany) of insect electrical signal, and then connected to the 4-channel IDAC acquisition controller (IDAC4, Syntech, Germany). Therefore, the LFP signals at the CX of the honeybee can be recorded and observed in real-time through computer software ‘Autospike’ (Synthch, Germany). 17 Honeybees were used for signal acquisition and 10 signals were recorded from every sample honeybee.

### Frequency Bands Identification Method

There are obvious differences in the frequency bands of brain electrophysiological signals among different species. In addition, the dominant rhythm in different species also exhibits certain differences. For example, the alpha rhythm is the dominant rhythm in the human scalp Electroencephalogram (EEG) while theta rhythm is in the hippocampus of lower mammals (Lopes da Silva 1992). Obviously, it is inappropriate to apply the frequency band definition to honeybees directly. According to the principles of frequency bands definition in previous research (Klimesch 1999), we analyzed the LFP signals collected at the CX of honeybees during the calm and flapping period by the power spectrum density method, and then proposed the frequency bands of honeybees in this case.

Before analyzing the LFP signals to identify frequency bands, preprocessing procedures of noise reduction and baseline drift correction were first applied in Matlab (The MathWorks, Natick, MA). The LFP signals are decomposed by the wavelet packet decomposition method to the minimum frequency band of the last layer less than 1 Hz. The minimum frequency band is removed. Then, the signals are reconstructed by the wavelet packet reconstruction method. Thus, the LFP signals with baseline drift removed are obtained. Signal denoising was achieved by the wavelet soft threshold denoising method and baseline drift correction was by the decomposition and reconstruction of wavelet packets. After being preprocessed by those methods, baseline drift and noise interference were improved without affecting the results of the following analyses.

### Feature Extraction Methods

#### Power Spectral Density (PSD)

Burg algorithm was performed to calculate the PSD of the LFPs. The calculation principle is to minimize the sum of the power of the forward and backward prediction errors of the sequence (Burg 1975). Burg algorithm has low computational complexity and high resolution for PSD calculation. Matlab provides a built-in command called *pburg.m* function to calculate the PSD. In this study, the AIC criterion was used for estimating the order of the AR model (Akaike 1973). Then, the PSD estimate was obtained by the function.

#### Wavelet Packet Decomposition

Wavelet analysis projects the signal in the space formed by a series of wavelet basis functions, thus realizing multiresolution analysis (Mallat 1988). However, wavelet analysis only describes the low-frequency part of the signal. In this study, high-frequency parts of the LFPs also contain a lot of detailed information. The wavelet packet can further decompose the wavelet space and also decompose the high-frequency part of the signal (Coifman et al. 1994). Thus, wavelet packet analysis was used for decomposing the information of the signal into sub-bands without redundancy. The LFPs are decomposed by the wavelet packet with a certain layer and a series of coefficients of the wavelet packet are obtained. The square sum of the coefficients is defined as the wavelet packet energy (Equation 1).


$$\begin{array}{l} E_{j}=\underset{i=1}{\overset{n}{\sum }}\;∥c_{i}{∥}^{2} \end{array}$$ (1)

Where *j* indicates the number of the frequency band; $E_{j}$ is the wavelet energy of the frequency band; *n* is the number of the data points; and $c_{i}$ is the wavelet packet coefficient.

#### Lempel-Ziv Complexity (LZC)

LZC measures the number of new occurring patterns for the time sequences (Lempel and Ziv 1976). The time sequence was converted into a binary sequence by comparing with a threshold. In this study, the threshold is the mean value of the sequence. LZC is obtained by normalizing the number of the new occurring patterns of the binary sequence (c) with the upper bound of c.

## Results

### Frequency Bands Identification and Oscillation Components

**Fig. 2.**
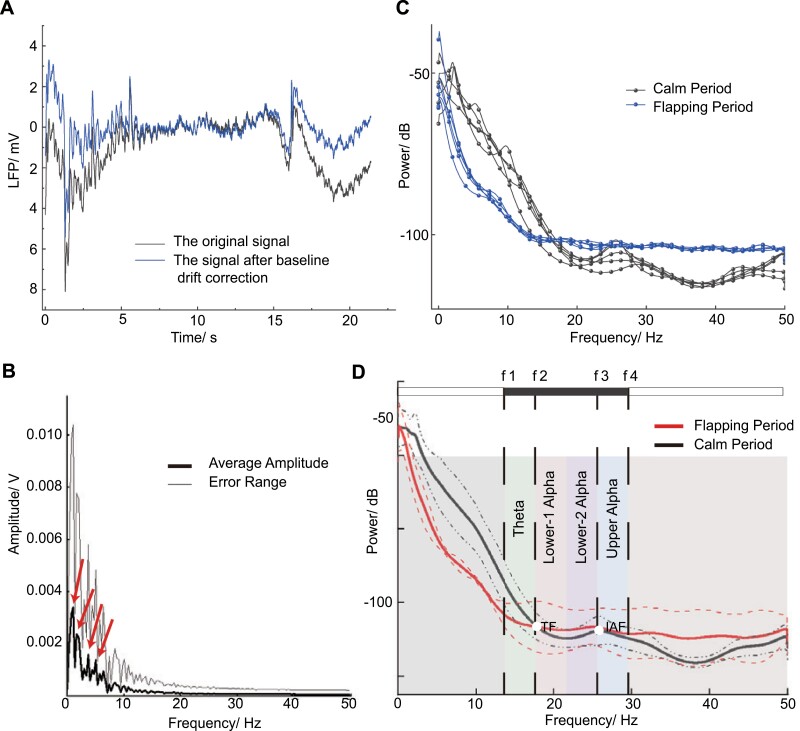
Frequency bands and oscillations identification. (A) Comparison between the original LFP signal (black line) and the LFP after baseline drift correction (red line). (B) From the results of the amplitude spectrum, four oscillation components were identified, which were indicated by red arrows. All oscillation components were below 10 Hz, which belonged to delta wave. (C) Five PSD evaluation samples in each of the two periods (blue line indicates the calm state and black line indicates the flapping state). (D) Frequency bands of theta and alpha were identified by TF and IAF points. TF was identified by the transition from theta synchronization to alpha desynchronization. IAF was identified by the peak of power spectra of the calm period. The alpha wave included lower-1, lower-2, and upper alpha. The red dashed lines represented error ranges of the flapping period and the black dashed lines represented error ranges of the calm period.

According to the baseline drift correction method mentioned above, the DC and low-frequency components in the original signals are removed. Thus, the possible distortion in the power spectrum analysis is prevented. An example of the LFP signal during a flapping period before and after baseline drift correction is shown in Fig. 2A. According to the event-related desynchronization theory, compared with a calm period, alpha power decreases while theta power increases in a testing period (Pfurtscheller 1992). The power of the LFP signals of honeybees during a flapping period and calm period was calculated. The power spectrum results of five samples in each of the two periods are shown in Fig. 2C. The overall average result is shown in Fig. 2D. The power of the LFP signals of honeybees during a flapping period and calm period were calculated, as shown in Fig. 2A. To identify the theta and alpha rhythms, two crucial points were required to recognize. In the plot of the power spectra, the transition frequency (TF) point ($f_{2}=18Hz$) is identified by the transition from theta synchronization to alpha desynchronization. And the individual alpha frequency (IAF) point ($f_{3}=26Hz$) was identified by the peak of the power spectra during a flapping period. As the identification methods mentioned above, the threshold between theta and delta rhythm was identified as $f_{1}=14Hz$, and the upper boundary of upper alpha was $f_{4}=30Hz$. Lower alpha rhythm was broken into two sub-bands called lower-1-alpha and lower-2-alpha, which were considered to occupy the equal bandwidth. Therefore, four primary rhythms in the CX of honeybees were all identified. Specifically, theta is 14–18 Hz, lower-1-alpha is 18–22 Hz, lower-2-alpha is 22–26 Hz, and upper alpha is 26–30 Hz.

After frequency bands were identified, some oscillation components were recognized by spectral analysis as shown in Fig. 2B. Delta Rhythm was dominant during honeybees’ spontaneous flapping behaviors, which was quite different from humans in that delta rhythm was dominant mainly during sleep. Specifically, delta oscillation was mainly manifested in 1 Hz, 1.5 Hz, 3.5 Hz, 5.0 Hz, and 6.4 Hz, where peaks occurred in frequency spectral. Those results indicated that a kind of segmental delta oscillation in analogy to segmental distribution in CX might relate to honeybee’s spontaneous flapping behaviors not in complex environments.

### Energy Distribution

**Fig. 3.**
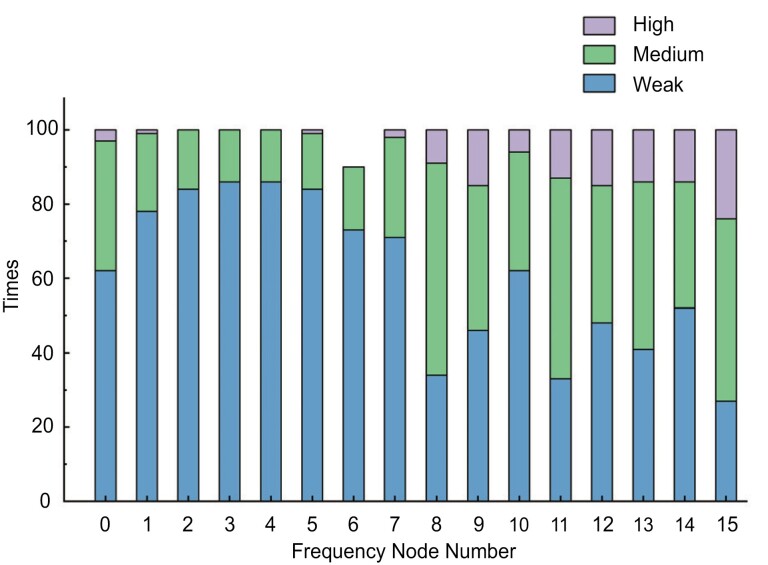
Correlation degree distribution results of 16 nodes after 4-level wavelet packet composition. The higher the times of weak, the greater the difference between the flapping and calm period in the frequency node. Thus, in frequency nodes of 2, 3, 4, 5, the occurrence times of ‘weak’ were all over 80. In the above frequency bands, it showed a relatively great difference between the two periods.

In order to obtain the energy distribution results in different frequency bands, wavelet packet composition was first applied at four layers. During this process, a series of wavelet packet coefficients were obtained. In the last layer, the whole frequency band (50 Hz) is decomposed into 16 frequency bands with the same bandwidth of 3.125 Hz by the wavelet packet method. According to the center frequency from low to high, those frequency bands are defined as node [4,1], node [4,2] …, and node [4,16]. To verify whether the wavelet packet coefficients could be an identification factor, we extracted the wavelet packet coefficients at the last layer during the honeybees’ flapping period and calm period. The Kendall coefficient is used for measuring the degree of correspondence between the two states and assessing the significance of this correspondence (Kendall 1938). Then Kendall coefficients between the wavelet packet coefficients of the two states at 16 frequency bands (each of 16/50 Hz) were calculated respectively. For example, the Kendall coefficient between the wavelet coefficient of the node [4,1] during the calm state and the node [4,1] during the flapping state was calculated as one of the results of node [4,1]. The harmonious correlation degree between the two states was divided into three levels, which were weak (0–0.3), medium (0.3–0.6), and high (0.6–1). Obviously, the lower the harmonious correlation between the two states, the greater the difference between the flapping period and calm period in a specific frequency band. Thus, those frequency bands with lower harmonious correlation could be identified as the recognition bands and were considered to relate to flapping behaviors to some extent. As shown in Fig. 3, there was a higher occurrence frequency of ‘weak’ (times > 80) in the frequency node of [4,2], [4,3], [4,4], [4,5], i.e., 6.25–18.75 Hz. This frequency band concluded a part of delta wave and all theta wave. Combined with the analyses mentioned above, those frequency bands were most likely to be highly correlated with honeybee’s spontaneous flapping behaviors.

**Fig. 4.**
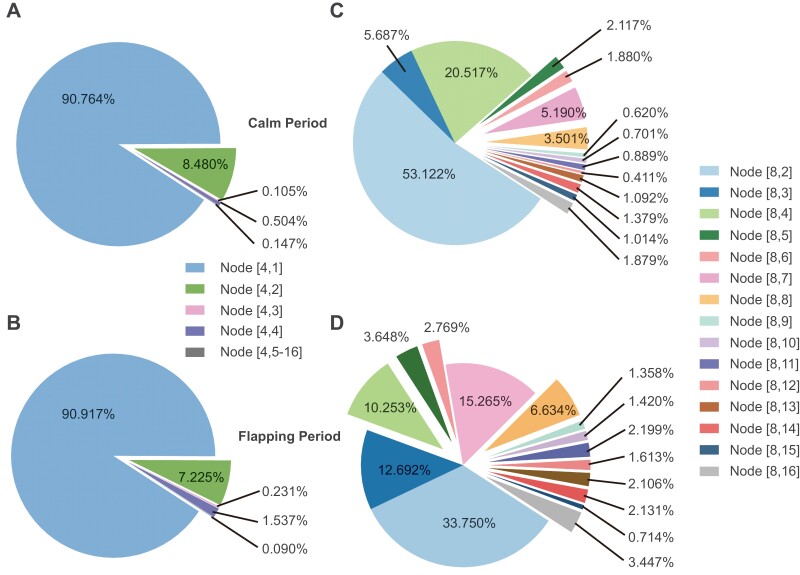
Energy distributions of 16 frequency bands in 50 Hz and 2-16 nodes of 256 frequency bands in 50 Hz. (A) The energy distribution results of 16 frequency bands in 50 Hz during the calm period. Most energy was distributed in frequency node 1. (B) The energy distribution results of 16 frequency bands in 50 Hz during the flapping period. The distribution was not much different from the results during the calm period. (C, D) The frequency node 1 was divided into 16 nodes in order to further observe the distribution in node 1 of (A, B). The bandwidth of each frequency node was 50/256 Hz.

Finally, as shown in Fig. 4A and B, the energy distribution result of 16 frequency nodes during the calm and flapping period was obtained. Obviously, most of the energy was distributed in node [4,1], and the second was node [4,2]. Extremely low energy was distributed in high-frequency bands. In order to further analyze the energy distribution of node [4,1], 8-layer wavelet packet decomposition was applied and each frequency bandwidth was 50/256 Hz. Because in 8-layer decomposition, node [8,1] only contained invalid information of extremely low frequency, only the energy distribution result of nodes [8,2] to [8,16] was calculated as shown in Fig. 4C and D. The energy was mainly distributed in node [8,2], [8,3], [8,4], whether it is during a flapping or calm period. Compared to the results of the calm period, the frequency bands where the energy distribution was significantly reduced were node [8,2] and node [8,4], while increased were node [8,3], node [8,7], and node [8,8] during the flapping period. In addition, there were increments in energy ratios in all relatively higher bands. Those results indicated that honeybee’s spontaneous flapping behaviors were probably highly correlated with the collaboration of inhibition of some specific sub-bands activities such as node [8,2], [8,4], and enhancement of some specific sub-bands activities such as node [8,3], [8,7], [8,8], and other higher sub-bands in delta wave.

### Nonlinear Characteristics

**Fig. 5.**
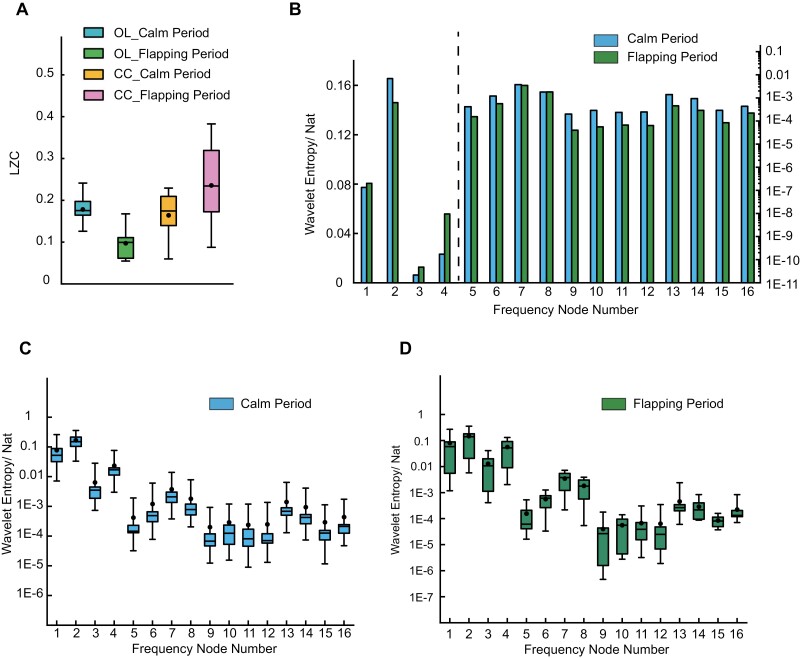
Nonlinear analysis results during the calm and flapping period. (A) LZC results during the calm and flapping period of OL and CX. (B) The wavelet entropy values of 16 frequency nodes in 50 Hz during the calm and flapping period. The difference among the entropy values of 5–16 nodes was relatively small, thus the logarithmic coordinates were used. The dashed line represented the boundary. (C, D) The wavelet entropy results of CX of 16 frequency nodes in 50 Hz during the calm period (C) and flapping period (D).

Due to the uncertainty and complexity characteristics of neural activity, applying nonlinear analysis to those LFP signals was valuable. In order to obtain the changes in functional states of CX during the flapping period, we first calculated the Lempel-Ziv Complexity (LZC) on the whole frequency scale. Considering that during a flapping period, the whole brain of the honeybee may be relatively active compared with a calm period, those effects should be excluded. Thus, a series of LFP signals recorded from the optic lobe (OL) during the flapping period was used for comparison, because OLs are considered to be primarily involved in the visual pathway. The LZC results were shown in Fig. 5A. Compared to the LZC values of CX during the calm period, the average value during the flapping period increased by 43.6%. It was obvious that when the spontaneous flapping behavior occurred, the neuron clusters activity of CX was more random and contained more information. Also, compared with the calm period, functional mode changes of neuron activity in CX were faster during the flapping period. In addition, LZC values in OL during the flapping period were smaller whether compared with the calm period in OL or the flapping period in CX. This perhaps indicated that when spontaneous flapping behaviors occurred, some visual neural pathways were inhibited at the same time. Since the effect of the possible increase in overall brain activity during the flapping period was excluded, it could be concluded that CX was involved in honeybee’s spontaneous flapping behavior.

Furthermore, in order to explore the changes in nonlinearity of each rhythm respectively, wavelet entropy analysis (4-layer, 16 nodes) was applied to LFP signals during the flapping and calm period. In general, wavelet entropy values of frequency nodes 1–4 were much larger than nodes 5–16 (Fig. 5B). For this reason, the dashed line in Fig. 5B was used as a boundary line. The linear coordinate was used for frequency nodes 1–4, logarithmic coordinate for frequency 5–16. This indicated that functional modes changed faster in delta wave than in higher frequency bands, which also confirmed the conclusion mentioned in the energy distribution analysis. As shown in Fig. 5C and D, wavelet entropy results both fluctuated a little, but compared with the calm period, more fluctuations were manifested during the flapping period. The LFP sequences of CX when the honeybee was flapping showed relatively higher randomness.

For frequency nodes 1–4, only node 2 showed a significant entropy decrease after the spontaneous flapping behavior happened, and node 1, 3, 4 all showed entropy increase to some extent. Specifically, node 4 (frequency band: 9.375–12.5 Hz) showed the fastest new functional modes generation speed. This indicated that high-frequency delta wave perhaps played a crucial role in the participation of CX during a spontaneous flapping period. For frequency nodes 5–16, entropy values all showed different degrees of decrease during the flapping period, but it was not significant. In conclusion, complexities of the new functional modes change of CX in theta and alpha wave were relatively low when the spontaneous flapping behavior occurred.

## Discussion

It has been a long-standing question that which parts of the brain participate and play a crucial role in honeybee’s flight. Although many works have explored the neuro-mechanism of the comprehensive decision-making process during flight navigation and onset-offset behaviors, how neural activity modes change during spontaneous rhythmic flapping behavior is still unclear. Thus, we approach this problem from the perspective of brain activity changes and rhythm oscillation.

In previous research, the hypothesis that CX is involved in insects’ motor control was speculated by behavioral experiments. If this hypothesis can be confirmed from the perspective of neural activities, it would be helpful to further understand the flight behavior of insects. Our results suggest that delta wave of CX played a primary role during the honeybee’s rhythmic flapping without a decision-making process. In EEG of humans, delta wave is dominant mainly during sleep. By analogy to this conclusion, honeybee’s rhythmic flapping behavior without decision-making is perhaps a kind of spontaneous locomotor activity, that is, an autogenesis behavior that evolved during some evolution stages of honeybees.

In order to confirm whether CX plays a role in spontaneous flapping behavior, OL was selected as a reference brain subregion to exclude the interference of the whole brain activity changes. There was an interesting phenomenon that neuron clusters of OLs instead showed inhibition of neural activities during the spontaneous flapping period. About this phenomenon, we could speculate that honeybees might inhibit the activities of neural pathways that are not related to the current flapping behavior to save energy and allocate energy rationally. Anyway, compared with OLs, neural clusters of CX significantly showed a more complex functional change mode and faster new mode generation speed. Therefore, CX does participate in the honeybee’s spontaneous flapping behavior. Although here we could not confirm whether the role CX plays in honeybee’s spontaneous flapping behavior is primary or not, this conclusion provides theoretical support in the aspect of neuro-mechanisms for the selection of stimulating points of insect cyborgs. One of the primary goals of developing a honeybee cyborg is to be able to artificially control the occurrence of its flight behavior. Previously, the selection of the stimulus parameters for honeybee cyborgs was mostly based on experience and lacked theoretical support. From our results of the sub-band energy ratio, the frequency range where the energy ratio changes sharply is the preferred choice for the voltage frequency used to stimulate honeybees to flap wings. The frequency bands with significant changes in LZC and wavelet entropy in the calm and flapping states are the same. These rhythms are more correlated with the changes in the functional states of the brain when wing flapping occurs. This may open new avenues for a finer control approach.

Our experimental results of physiological signatures of brain activity changes during wing flapping will help to elucidate the functional state of brain rhythmical activities associated with wing flapping. It is also applicable to improve the behavioral control accuracy of insect cyborgs.
